# Supplementary material for: Coronary Artery Bypass Grafting Versus Percutaneous Coronary Intervention for Left Main Coronary Artery Disease—Long-Term Outcomes
Source: J Clin Med. 2025 Aug 14;14(16):5747. doi: 10.3390/jcm14165747 (PMC12387798; doi:10.3390/jcm14165747)
Supplement: Supplementary file 1 [file jcm-14-05747-s001.zip › jcm-3740529-supplementary.pdf]

TABLES

Table S1. Baseline clinical and angiographic characteristics in the propensity-score–matched cohorts.

	Propensity-score–matched patients	
	**CABG (53)**	**PCI (53)**
**CLINICAL CHARACTERISTICS**		
**Age, years; median (Q1;Q3)**	70.8 (61.8;75.7)	71.0 (61.0;76.0)
**Gender, male; n(%)**	41 (77.4)	42 (79.2)
**BMI, kg/m2; median (Q1;Q3)**	28.2 (26.9; 29.1)	28.1 (26.7;28.8)
**Frailty; n(%)**	4 (7.5)	4 (7.5)
**Current smoking; n(%)**	13 (24.5)	12 (22.6)
**COPD; n(%)**	9 (17.0)	9 (17.0)
**Diabetes; n(%)**	20 (37.7)	19 (35.8)
**with insulin; n(%)**	9 (17.0)	8 (15.1)
**Hypertension; n(%)**	44 (83.0)	46 (86.8)
**Severe PH; n(%)**	7 (13.2)	7 (13.2)
**Dyslipidemia; n(%)**	39 (73.6)	39 (73.6)
**Congestive heart failure; n(%)**	10 (18.9)	11 (20.8)
**LVEF, %; median (Q1;Q3)**	34.1 (31.2;50.1)	33.7 (30.8; 49.6)
**CKD; n(%)**	15 (28.3)	16 (30.2)
**Atrial fibrillation; n(%)**	13 (24.5)	13 (24.5)
**Anemia; n(%)**	15 (28.3)	17 (32.1)
**Prior MI; n(%)**	27 (50.9)	28 (52.8)
**Prior revascularization; n(%)**	19 (35.8)	19 (35.8)
**Indication; n(%)**		
**CCS**	22 (41.5)	20 (37.7)
**ACS**	31 (58.5)	33 (62.3)
**STEMI**	1 (1.9)	2 (3.8)
**NSTEMI/UA**	30 (56.6)	31 (58.5)
**PAD; n(%)**	5 (9.4)	5 (9.4)
**Prior stroke/TIA; n (%)**	7 (13.2)	8 (15.1)
**Active cancer; n(%)**	0 (0.0)	3 (5.7)
**EuroSCORE II, %; median (Q1;Q3)**	3.8 (3.3;5.2)	3.9 (3.1;5.5)
**STS score, %; median (Q1;Q3)**	2.5 (2.2;3.2)	2.6 (2.0;3.4)
**ANGIOGRAPHIC PARAMETERS**		
**Qualifying LM lesion**		
**LM coronary segment**	52 (98.1)	51 (96.2)
**LM equivalent disease**	1 (1.9)	2 (3.8)
**Bifurcation or trifurcation disease of distal LM; n(%)**	46 (86.8)	44 (83.0)
**LM considered as culprit lesion in ACS, n(%)**	5/31 (16.1)	6/33 (18.2)
**Affected non-LM arteries, n(%)**		
**0**	9 (17.0)	10 (18.9)
**1**	13 (24.5)	14 (26.4)
**2**	18 (34.0)	17 (32.1)
**3**	13 (24.5)	12 (22.6)
**Severe calcification; n(%)**	19 (35.8)	18 (34.0)
**Total occlusion; n(%)**	13 (24.5)	13 (24.5)
**SYNTAX score; median (Q1;Q3)**	34.1 (29.2;41.4)	34.0 (28.9;41.2)
